# Response of Tomato Fruit Quality Depends on Period of LED Supplementary Light

**DOI:** 10.3389/fnut.2022.833723

**Published:** 2022-01-31

**Authors:** Shuya Wang, Ning Jin, Li Jin, Xuemei Xiao, Linli Hu, Zeci Liu, Yue Wu, Yandong Xie, Wen Zhu, Jian Lyu, Jihua Yu

**Affiliations:** ^1^College of Horticulture, Gansu Agricultural University, Lanzhou, China; ^2^State Key Laboratory of Aridland Crop Science, Gansu Agricultural University, Lanzhou, China

**Keywords:** tomato, LED, period, flavor quality, nutrition quality

## Abstract

Light is an important environmental factor that regulates the activity of metabolism-related biochemical pathways during tomato maturation. Using LED to improve lighting conditions during the process of tomato growth and development is a feasible and efficient method to improve the quality of tomato fruit. In this study, red and blue LEDs were used to supplement light on “MicroTom” tomato plants for different periods of time in the morning and evening, and the differences between the primary and secondary metabolites and other nutrient metabolites in the tomato fruit were analyzed using liquid chromatography and liquid chromatography mass spectrometry and other methods. Supplementing light in the morning promoted the accumulation of vitamin C, organic acids, amino acids, carotenoids, phenolic acids, and other health-promoting substances in the tomato fruits. Supplementing light in the evening significantly increased the content of sugars, flavonoids, and aromatic substances in tomato fruits, whereas the promoting effect of LED on the accumulation of amino acids and carotenoids was lower in the evening than in the morning. Both morning and evening light supplementation reduced the mineral content of fruit. In conclusion, morning light supplementation improved the nutritional quality of tomato fruits, while evening light supplementation improved their flavor.

## Introduction

Tomatoes are one of the most widely cultivated vegetables in the world, and are deeply loved for their unique flavor and rich nutritional quality. Tomatoes contain soluble sugars, organic acids, polyphenols, carotenoids, amino acids and other nutrients (1). Eating tomatoes can prevent cancer and other diseases and have great health benefits (2, 3). The ever-increasing population has led to the pursuit of high yields of tomatoes. In order to increase yields, breeders have to lower the requirements for tomato quality and discard some good quality traits and varieties (4, 5). In addition, factors such as climate change, intensification of environmental pollution and improper use of fertilizers have led to the deterioration of tomato quality (6, 7). In recent years, with the improvement of people's requirements for food quality, it is generally believed that tomatoes have lost their “childhood taste,” and the goal of increasing the quality of tomato fruits has attracted more and more attention.

Previous studies have found that there are many ways to improve tomato fruit quality. Appropriate fertilization and irrigation methods can promote the nutritional quality of tomato fruits (8–10). Root application or foliar spraying of certain concentrations of exogenous substances such as methyl jasmonate, quercetin, selenium, and potassium can increase the contents of sugars, carotenoids, and other nutrients in tomato fruits (1, 11–13). Small molecular gases such as nitric oxide and hydrogen sulfide also significantly improved the nutritional and sensory quality of tomato fruits (14, 15).

Light is an important environmental factor that regulates the activities of metabolite-related biochemical pathways during tomato ripening and plays an important role in determining tomato quality (16). Both the leaves and fruits of tomato contain photoreceptors, including phytochrome and cryptochrome (17, 18), and appropriate illumination can promote the absorption of light and enhance the photosynthetic capacity of tomato (19). Moreover, light can contribute to promoting the absorption and transport of water and nutrients (20), thereby influencing metabolic processes and metabolite production (21). Light-emitting diodes (LEDs) are an efficient environmentally friendly source of artificial light, the supplementary illumination provided by which can promote the growth and development of tomatoes and other vegetables (22, 23), and the findings of recent studies have revealed that such supplementary light can play an important role in improving tomato fruit quality. LEDs with different spectra and supplementary LED illumination of differing durations can have different effects on tomato fruit metabolites and contribute to enhancing the amounts of key compounds related to tomato fruit quality characteristics (16, 24). For example, compared with white light, a combination of red and blue (R:B, 3:1) LED illumination has been shown to increase the contents of soluble solids, glucose, fructose, and sucrose in tomato fruits by promoting the accumulation of proteins related to glucose metabolic pathways (25), whereas compared with lighting using high pressure sodium lamps, LEDs have been found to significantly increase the content of lycopene in tomato fruits (26). Similarly, a combination of red and blue LEDs has been demonstrated to promote lycopene biosynthesis and the accumulation of carbohydrates by increasing the contents of melatonin in tomato fruits (27), whereas either blue or a combination of red and blue LEDs was found to increase the contents of potassium and beta carotene in fruits by promoting the absorption and transport of potassium by tomato roots (28). Furthermore, it has been observed that blue light LEDs can significantly increase the contents of soluble solids, lycopene, and phenolic compounds in post-harvest tomato fruits (29), whereas high-intensity blue light LEDs can stimulate the antioxidant system in tomato fruit, thereby enhancing the ascorbic acid content (30). Continuous illumination with red light LEDs has also been found to significantly promote increases in the contents of lycopene, beta carotene, phenolic acid, and flavonoids in post-harvest tomato fruits (31), whereas significant increases in the contents of free amino acids in post-harvest tomato fruits have been detected in response to continuous illumination with blue light LEDs (32). In addition, the provision of red light LED lighting can improve the content of Mg, Ca, Cu, and other mineral elements in tomato fruits (19), and the sensory qualities of aroma and texture in fruits can be significantly enhanced in response to an increase far-red irradiation (33). In our previous studies, we have also found that exposing tomato plants to red-blue light LEDs can improve the soluble sugar and soluble protein contents of fruits (34).

Collectively, the findings of these studies indicate that appropriate LED lighting can contribute to enhancing the quality of tomato fruit. However, although previous studies in this regard have tended to focus on the quality and intensity of LED light, as well as the duration of supplementary illumination, it is also well-established that the metabolic processes of plants are regulated by circadian rhythms and biological clocks (35, 36). Consequently, it might be predicted that the effects of supplementary LED illumination on tomato quality would be dependent on an appropriate timing of light supplementation. It is thus considered necessary to examine the temporal effects supplementary LED lighting on the quality of tomato fruit. At present, however, the responses of tomato fruit to the provision of supplementary light at different times during the day have yet to be sufficiently well-characterized. Accordingly, in this study, we examined the effects supplementary light treatments with red and blue LEDs provided at different periods of time on tomato, with a specific focus on differences between primary and secondary metabolites and other nutritional metabolites, analyzed based on liquid chromatography and liquid chromatography mass spectrometry and other approaches. The principle objectives of study were to clarify the effects of the timing of LED supplementation on tomato fruit quality, provide new insights for enhancing the quality of tomato fruit quality, and establish a new theoretical basis for supplementary LED lighting technology for tomatoes.

## Materials and Methods

### Plant Materials and Growing Conditions

The tomato variety used in this experiment was “Micro Tom.” After soaking in warm water, tomato seeds were placed in a dark artificial climate box at 28°C to promote germination. After 36 h, the uniformly germinated seeds were sown on the seedling substrate, and then put into the artificial climate box where the seedlings were raised. The conditions of the artificial climate chamber were 12 h of illumination and 12 h of darkness. When illuminating, the temperature was 26°C, the humidity was 70%, and the light intensity was 450 μmol·m^−2^·s^−1^. Under dark conditions, the temperature was 18°C and the humidity was 50%. When the seedlings had two leaves and one heart, they were moved into the greenhouse for normal management.

### Experimental Design

**Figure 1:**
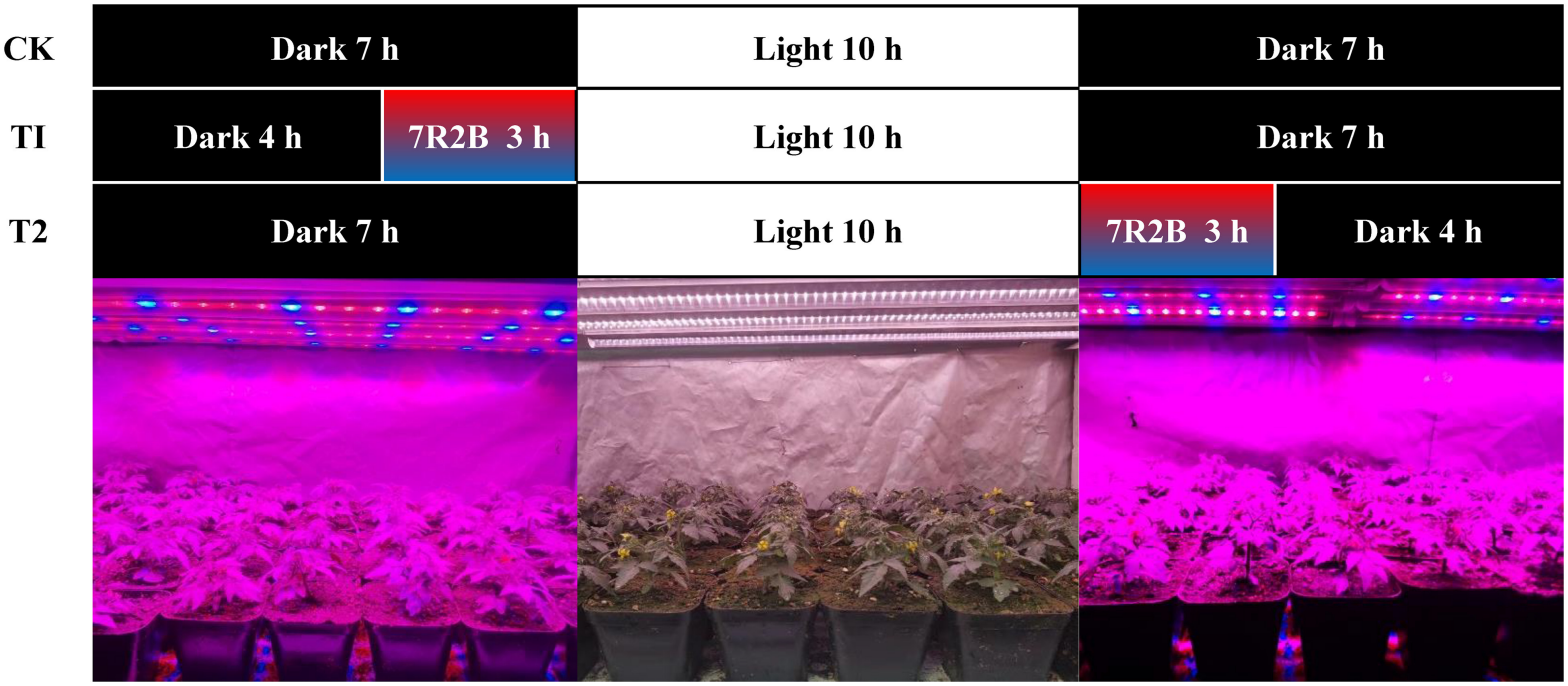
Schematic diagram of the illumination time of each treatment. CK, no light supplementation control; T1, light supplementation for 3 h in the morning; T2, light supplementation for 3 h in the evening.

The experiment was carried out in a plant light supplement cultivation frame completely isolated from natural light in the greenhouse. Two kinds of LED light sources were installed on the cultivation rack: one was a full-spectrum LED plant growth light (T8-0.9M-28W-220V, Xiamen rural Hui Photoelectric Technology Co., Ltd.), which provided 10 h of daylight between 8 A.M. and 18 P.M., with light intensity 400 μmol·m^−2^·s^−1^. The other was a red and blue (7R2B) LED plant growth light (HY-85CM-27 × 3W-RB, Shenzhen Houyi Energy Saving Tech Co., Ltd.), which was the supplementary light source in this experiment, with light intensity 51 μmol·m^−2^·s^−1^. The experiment consisted of three treatments: treatment without supplementary light (CK), red and blue light supplementation for 3 h before 8 o'clock in the morning (T1), and red and blue light supplementation for 3 h after 18:00 in the evening (T2) (Figure 1). Fifty tomato plants with the same flowering time were selected for each treatment when the first flower was just blooming, and transferred to the plant light cultivation rack where the treatment commenced. When the tomato fruit matured, fruit of the same size and maturity were selected to determine the quality, and all the index analyses were set up with 3 biological repeats in each treatment.

### Sugar Components

Tomato fruit (5 g) were homogenized, transferred to a 25 mL volumetric flask with ultrapure water to make the volume constant, and then sonicated in a water bath at 30°C for 60 min, filtered into a 50 mL centrifuge tube at 4°C, centrifuge for 10 min at 10,000 r·min^−1^. Supernatant (2 mL) was extracted and filtered through a 0.22 μm aqueous filter, and the filtrate was used for liquid chromatography. The measuring instrument was a high performance liquid chromatograph (HPLC) with a differential refractive index display (Agilent 1100 Series, Agilent Technologies, USA), the chromatographic column was an LC-NH2 amino column (250 × 4.6 mm, Phenomenex, USA), the mobile phase was V(acetonitrile): V(water) = 75: 25, isocratic elution, flow rate 1.0 mL·min^−1^, column temperature 30°C, and the injection volume was 20 μL.

### Organic Acid Components

A fresh sample of tomato fruit (0.5 g) was transferred to a 25 mL volumetric flask with ultrapure water to make the volume constant, shaken well, transferred to a 50 mL centrifuge tube, centrifuged at 4°C, 10,000 r·min^−1^ for 10 min; 2 mL of supernatant was extracted and filtered through a 0.22 μm water system Filter by filter, and the filtrate was used for liquid chromatography (HPLC) determination. The measuring instrument was a high performance liquid chromatograph with a UV detector (Agilent 1260 Infinity II, Agilent Technologies, USA), the chromatographic column was X-Peonyx AQ-C18 (250 × 4.6 mm, FeiniGen Instrument, China), the detection wavelength was 210 nm, and the mobile phase was 0.2 mmol·L^−1^ dihydrogen phosphate sodium, isocratic elution, flow rate 1.2 mL·min^−1^, column temperature 30°C, injection volume was 5 μL.

### Amino Acid Components

Amino acid components were determined according to the method of Ma et al. (37) with slight modifications. The tomatoes were heated in a constant temperature drying oven at 105°C for 15 min, and then dried at 80°C to a constant weight. After grinding, 0.5 g was weighed into a 50 mL Erlenmeyer flask, 25 mL of 0.1% hydrochloric acid solution was added, then ultrasonically extracted for 15 min. The extract was transferred into a 50 mL centrifuge tube, centrifuged at 4°C, 10,000 r·min^−1^ for 10 min, the supernatant was filtered through a 0.22 μm aqueous filter, and the filtrate supplied for HPLC-MS (LC-MS) determination. The measuring instrument was a triple quadrupole LC/MS system (Agilent 1290 Infinity, Agilent 6460 Triple Quad, Agilent Technologies, USA), the chromatographic column was Poroshell 120 HILIC-Z (100 × 2.1 mm, Agilent Technologies, USA), and the mobile phase A was 20 mM ammonium formate (pH = 3): water = 1: 9, the mobile phase B was 20 mM ammonium formate (pH = 3): acetonitrile = 1:9, gradient elution (0 min, 100% mobile phase B; 11.5 min, 30% mobile phase A and 70% mobile phase B; 12 min, 100% mobile phase B), the flow rate was 0.5 mL·min^−1^, the column temperature 25°C, the injection volume 1 μL. The mass spectrometry ionization mode was the ESI positive ion mode, the drying gas temperature was 330°C, the gas flow rate 13.0 L·min^−1^, and the capillary voltage 1,500 V. The mass spectrometry detection parameters of each amino acid, such as parent ion and product ion, are detailed in Supplementary Table 1.

### Carotenoid Components

Carotenoid components were determined according to Kang et al. 's method (38) with slight modification. The tomato fruits were freeze-dried at −30°C for 72 h in a freeze dryer (Alpha 1-2 LDplus, Martin Christ, Germany). The freeze-dried tomatoes were ground into powder, weighed (0.5 g) into a 50 mL centrifuge tube, with 30 mL of petroleum ether and acetone mixture (2:1, v/v), then to a water bath for ultrasonic extraction. For carotene, the extract was collected in a brown bottle, and 30 mL of a mixture of petroleum ether and acetone was added until all the color was removed. The combined filtrate was transferred to a separatory funnel, washed twice with 250 mL of distilled water, the water phase was drained, and a small amount of anhydrous sodium sulfate was added to the upper extract to remove the water phase. The extract was filtered into a round-bottomed flask with a sand core funnel under vacuum and placed in a rotary evaporator at 40°C until dry. The dried extract was then dissolved with 25 mL of an acetonitrile: dichloromethane: methanol (55:20:25) mixture, and filtered through a 0.22 μm oil filter. The filtrate was used for liquid chromatography determination. The extraction process was protected from light. The measuring instruments were high performance liquid chromatograph (Alliance Waters e2695, Waters, USA) and ultraviolet detector, the chromatographic column was HPLC C18 (250 × 4.6 mm, Waters, USA), the detection wavelengths were 286 nm, 450 nm, 470 nm and 665 nm, and the mobile phase was V(acetonitrile): V(two Chloromethyl): V(methanol) = 55: 20: 25, isocratic elution, the flow rate 1.2 mL·min^−1^, the column temperature 30°C; the injection volume 10 μL.

### Phenolic Acids and Flavonoids

To determine the phenolic acids and flavonoids, 0.1 g of freeze-dried fruit powder was weighed and placed in a 5 mL centrifuge tube with 2 mL of methanol, and placed at 4°C for 1 h, shaking it 3 times during the hour. The tube was centrifuged at 8,000 r·min^−1^ at 4°C for 10 min, then 2 mL of supernatant was extracted, filtered through a 0.22 μm oil-based filter, and the filtrate was used for liquid chromatography measurement. The measuring instruments were high performance liquid chromatograph (Alliance Waters e2695, Waters, USA) and ultraviolet detector, the chromatographic column was HPLC C18 (250 × 4.6 mm, Waters, USA), the detection wavelength 240 nm, 280 nm and 322 nm, mobile phase A was methanol, mobile phase B was 1% acetic acid, gradient elution (Supplementary Table 2), flow rate 1.1 mL·min^−1^, column temperature 30°C, injection volume 10 μL.

### Mineral Element Content

To determine the mineral element content, 0.5 g of dried and ground tomato fruit was placed in a 150 mL erlenmeyer flask, and digested using the H_2_SO_4_-H_2_O_2_ digestion method. The digestion solution was washed into a 50 mL volumetric flask without damage, with ultrapure water to make the volume constant, and shaken well. The extract was used for the determination of mineral elements phosphorus (P) and potassium (K). 0.5 g of dried and ground tomato fruit was put into a porcelain crucible and ashing was carried out by dry ashing method. The ash was dissolved with 5 mL 6 mol·L^−1^ HCL, filtered and filled with water in a constant volume of 50 mL volumetric flask. The extract was used for the determination of mineral elements such as calcium (Ca), magnesium (Mg), copper (Cu), iron (Fe), manganese (Mn), zinc (Zn) and sodium (Na). Phosphorus was determined using the Mo-Sb colorimetric method. The K, Ca, Mg, Cu, Fe, Mn, Zn, and Na contents were determined by an atomic absorption spectrometer (ZEEnit700P, Analytik Jena AG, Germany).

### Volatile Compounds

To determine the contents of volatile compounds a sample of fresh tomato fruit (0.5 g), was added to 1.5 g anhydrous sodium sulfate, ground quickly and fully, and then pourrf into a headspace bottle. After closing the cover, the headspace bottle was placed on a magnetic stirrer, heated and stirred at 70°C for 10 min to balance the internal headspace gas, and then the electronic nose (PEN3, Airsense, Germany) was used to detect the volatile substances in the gas at the upper part of the headspace bottle. The detection conditions of the electronic nose were as follows: the flushing time was 60 s, the sensor zeroing time 5 s, the pre sampling time 5 s, the injection flow rate 400 ml·min^−1^, and the measurement time 120 s. Please refer to Supplementary Table 3 for the material type and performance description of the sensor.

### Statistical Analysis

There were replicates for each treatment and the results are presented as mean ± standard deviation (SD). One-way analysis of variance was performed, and Duncan's honesty significant difference test was used to assess the differences between two groups, using SPSS ver. 21 (SPSS, Inc., Chicago, IL, USA). Heat map production and hierarchical cluster analysis (HCA) were carried out in Origin 2021 (Origin, Inc., San Francisco, California, USA). HCA was based on the calculation of Pearson correlation. *P* < 0.05 were deemed statistically significant.

## Results

### Sugar Contents

**Figure 2:**
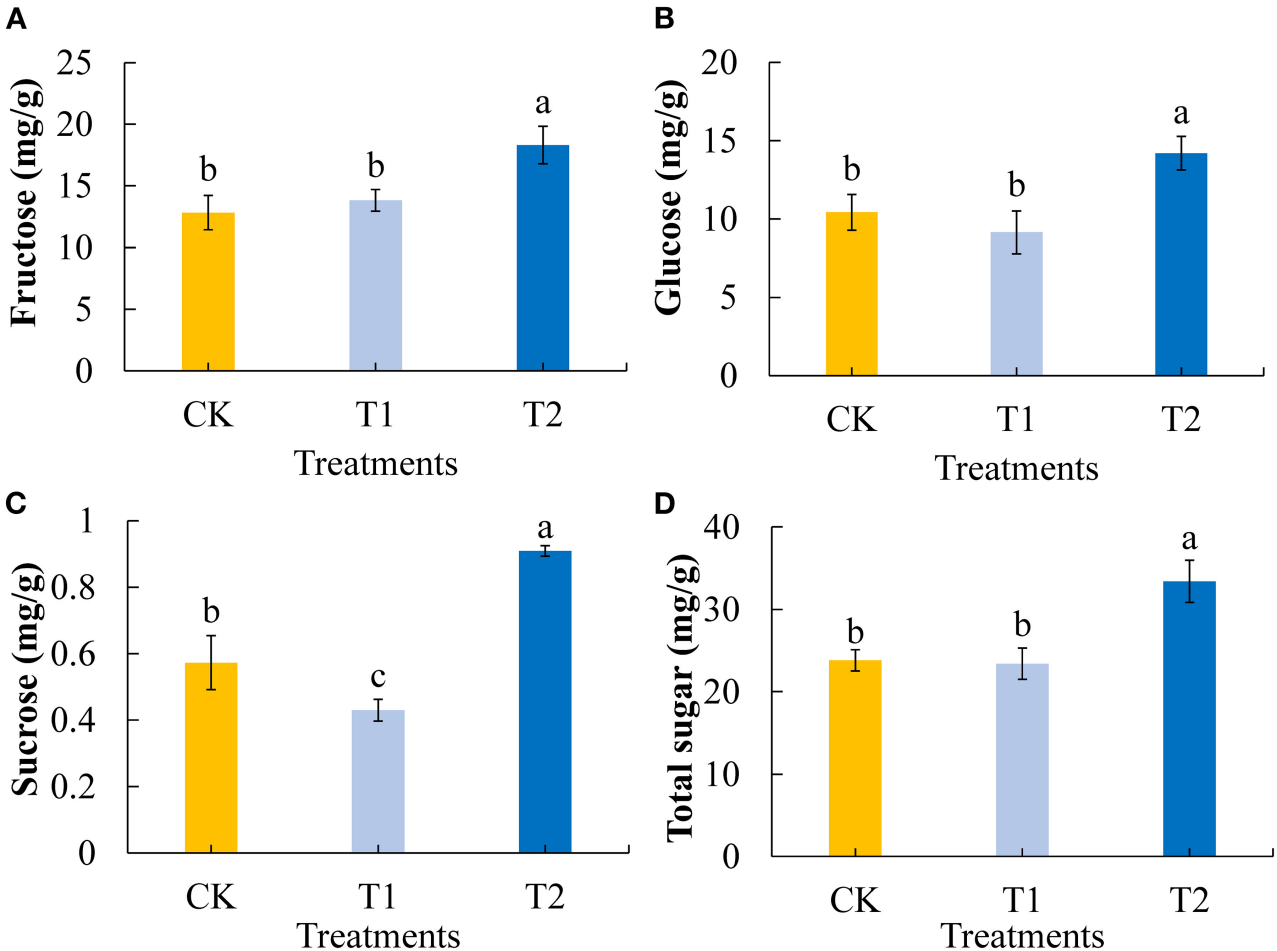
Fructose **(A)**, glucose **(B)**, sucrose **(C)**, and total sugar **(D)** contents in tomato fruits under different LED supplementary light periods. The data are expressed as average values ± SD (*n* = 3). ^a−*c*^Indicate significant differences between treatments (*P* < 0.05, Duncan's multiple range test). The bars indicate standard errors. CK, no light supplementation control; T1, light supplementation for 3 h in the morning; T2, light supplementation for 3 h in the evening.

Sugars are important plant constituents that contribute to the flavor of tomato fruit and play key roles in determining fruit quality (39). We first determined and analyzed the contents of the main soluble sugars (fructose, glucose, and sucrose) in tomato fruits. Compared with the control group, 3 h of evening light supplementation significantly increased the contents of fructose (Figure 2A) and glucose (Figure 2B) in the tomato fruits. Compared with the control group 3 h of morning light supplementation made no significant difference in the contents of fructose and glucose in the tomato fruits. Light supplementation in the evening significantly increased the sucrose content in tomato fruit, and conversely, light supplementation in the morning decreased the sucrose content (Figure 2C). Consistently, we found that the total sugar contents of tomato fruit were significantly increased by 3 h of evening light supplementation, although there was no significant difference between treatment and control fruits when supplementary light was provided in the morning (Figure 2D).

### Organic Acid Components

**Figure 3:**
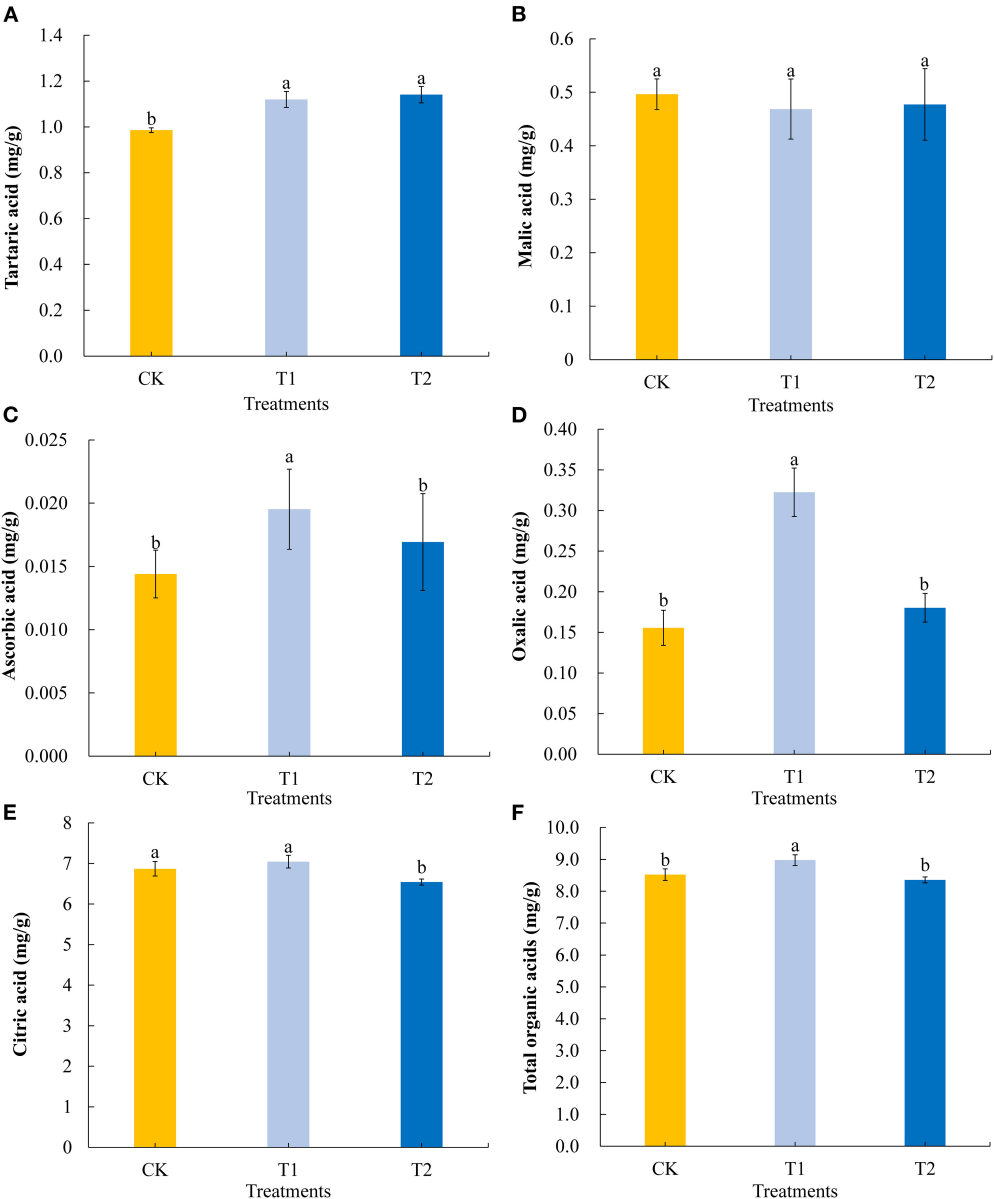
Tartaric acid **(A)**, malic acid **(B)**, ascorbic acid **(C)**, oxalic acid **(D)**, citric acid **(E)**, and total organic acids **(F)** contents in tomato fruits under different LED supplementary light periods. The data are expressed as average values ± SD (*n* = 3). ^a−*c*^Indicate significant differences between treatments (*P* < 0.05, Duncan's multiple range test). The bars indicate standard errors. CK, no light supplementation control; T1, light supplementation for 3 h in the morning; T2, light supplementation for 3 h in the evening.

Among the organic acids present in tomato fruits, we selected five to evaluate the differences in organic acid contents in tomato fruits in response to supplementary light at different times of the day. Compared with the control, 3 h of light supplementation in the morning and evening significantly promoted the accumulation of tartaric acid in tomato fruits (Figure 3A) but had no effect on the accumulation of malic acid (Figure 3B). Light supplementation for 3 h in the morning significantly promoted the accumulation of ascorbic acid (Figure 3C) and oxalic acid (Figure 3D) in the tomato fruits, while light supplementation for 3 h in the evening had no significant effect on the contents of ascorbic acid and oxalic acid. In contrast, exposing plants to supplementary light for 3 h in the evening promoted a significantly reduction in the accumulation of citric acid, whereas we detected no significant differences in the citric acid contents of treatment and control fruit when light supplementation was provided in the morning (Figure 3E). Light supplementation for 3 h in the morning significantly promoted the accumulation of total organic acids (Figure 3F) in tomato fruits, while light supplementation for 3 h in the evening had no significant effect on the accumulation of total organic acids.

### Amino Acid Contents

**Figure 4:**
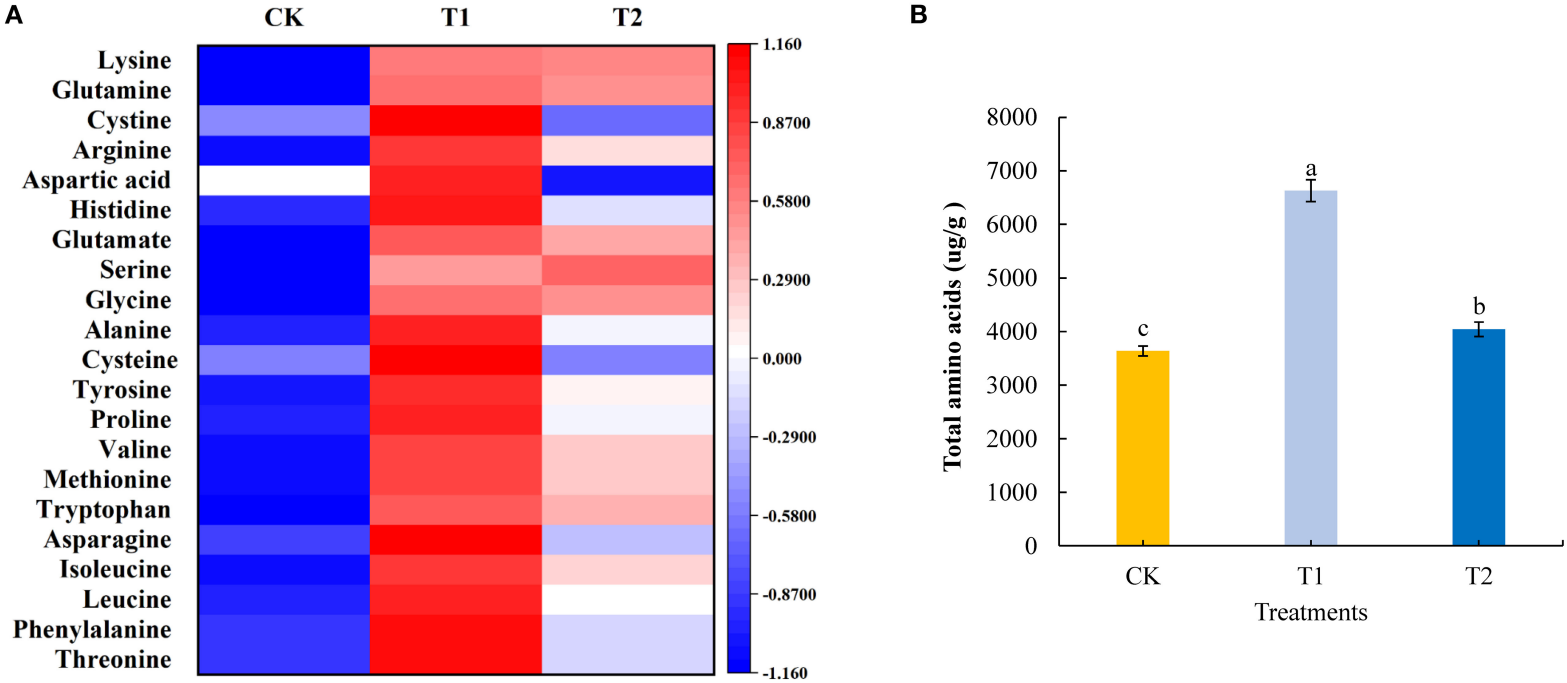
Amino acid component **(A)**, total amino acids **(B)** contents in tomato fruits under different LED supplementary light periods. The data are expressed as average values ± SD (*n* = 3). ^a−*c*^Indicate significant differences between treatments (*P* < 0.05, Duncan's multiple range test). The bars indicate standard errors. CK, no light supplementation control; T1, light supplementation for 3 h in the morning; T2, light supplementation for 3 h in the evening.

Amino acids are the basic substances that constitute proteins, maintain the normal metabolism of plants, and provide the material basis for life activities. In the present study, we quantitatively analyzed 21 amino acids in tomato fruits using liquid chromatography-mass spectrometry. We found that compared with the control treatment, with the exception of serine, for which there was no significant effect, supplementary lighting for 3 h in the morning significantly increased the contents of the assessed amino acids. Supplementing light for 3 h at night significantly increased the content of phenylalanine, leucine, isoleucine, tryptophan, methionine, valine, proline, tyrosine, alanine, glycine, glutamate, arginine, glutamine, and lysine in tomato fruits, significantly reduced the content of aspartic acid, and had no significant effect on the accumulation of cystine, histidine, serine, cysteine, asparagine, and threonine. Compared with the treatment of supplementing light for 3 h in the evening, supplementing light for 3 h in the morning significantly increased the accumulation of threonine, phenylalanine, leucine, isoleucine, asparagine, methionine, valine, proline, tyrosine, cysteine, alanine, histidine, aspartic acid, arginine, and cystine in tomato fruits (Figure 4A; see also Supplementary Table 4). Moreover, providing light supplementation for 3 h in both the morning and evening promoted significant increases in the accumulation of total amino acids in tomato fruits, with amounts in the fruit of plants exposed morning supplementation being significantly higher than those in plants receiving evening supplementation (Figure 4B).

### Carotenoid Components

**Figure 5:**
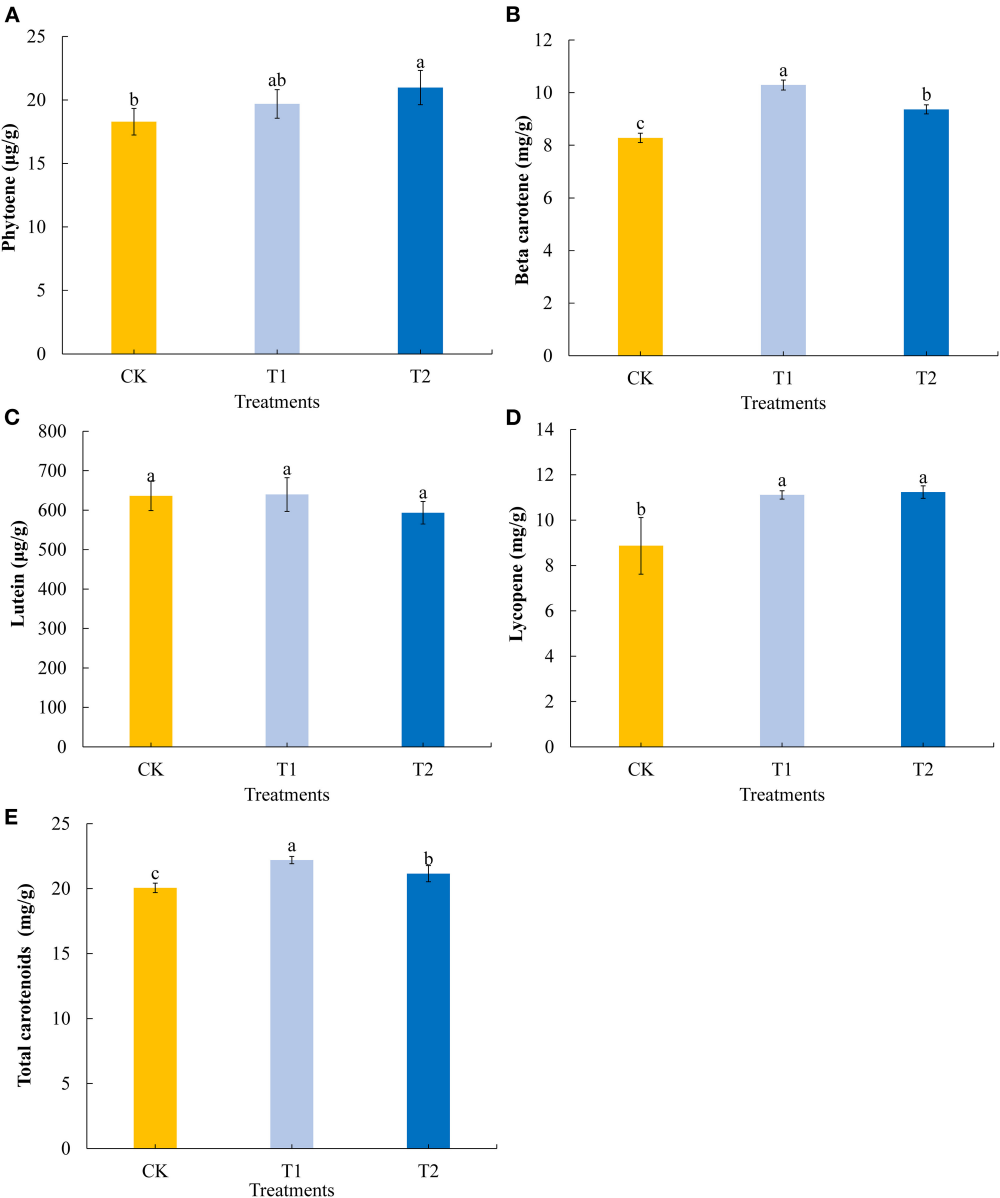
Phytoene **(A)**, beta carotene **(B)**, lutein **(C)**, lycopene **(D)**, and total carotenoids **(E)** contents in tomato fruits under different LED supplementary light periods. The data are expressed as average values ± SD (*n* = 3). ^a−*c*^Indicate significant differences between treatments (P < 0.05, Duncan's multiple range test). The bars indicate standard errors were indicated by bars. CK, no light supplementation control; T1, light supplementation for 3 h in the morning; T2, light supplementation for 3 h in the evening.

Carotenoids are important natural pigments in plants. We determined four common carotenoids in tomato fruits. We found that providing supplementary light for 3 h in the evening promoted a significant increase in the fruit contents of phytoene (Figure 5A), and that 3 h of both morning and evening light supplementation significantly increased the contents of beta carotene, with morning supplementation promoting a significantly higher accumulation of this carotene (Figure 5B). Both morning and evening light supplementation for 3 h had no significant effect on the lutein content in tomato fruits (Figure 5C), Both morning and evening light supplementation for 3 h significantly promoted the accumulation of lycopene (Figure 5D). Similarly, 3 h of both morning and evening light supplementation were found to promote significant increases in the accumulation of total carotenoids in tomato fruits, with morning supplementation having a more pronounced effect in this regard (Figure 5E).

### Phenolic Acids and Flavonoids

**Figure 6:**
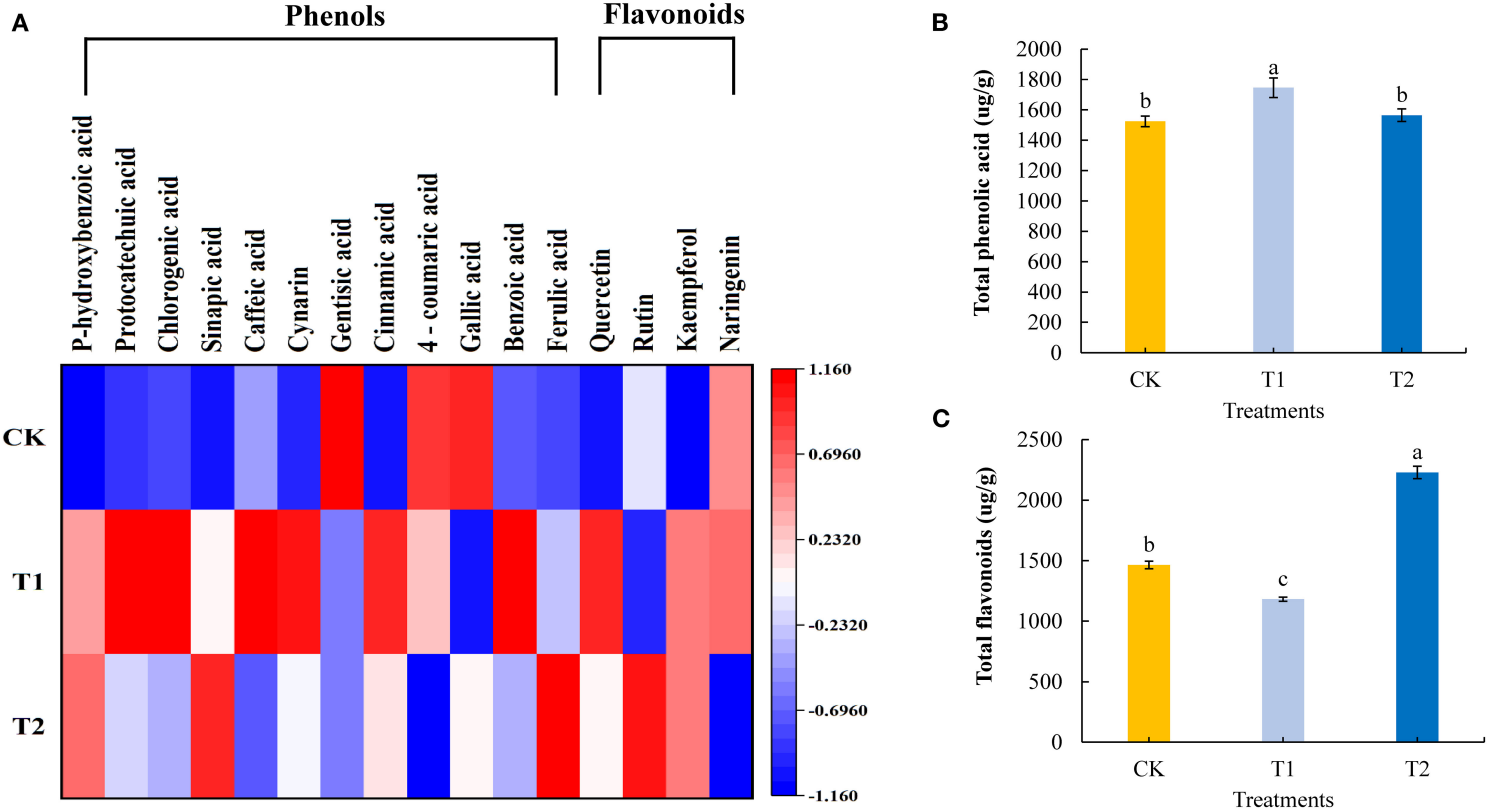
Phenolic acid component and flavonoids component **(A)**, total phenolic acid **(B)**, and total flavonoids **(C)** contents in tomato fruits under different LED supplementary light periods. The data are expressed as average values ± SD (*n* = 3). ^a−*c*^Indicate significant differences between treatments (*P* < 0.05, Duncan's multiple range test). The bars indicate standard errors. CK, no light supplementation control; T1, light supplementation for 3 h in the morning; T2, light supplementation for 3 h in the evening.

Phenolic acids and flavonoids are natural antioxidants in plants, which can prevent diseases in the human body. We quantitatively analyzed 12 phenolic acids and 4 flavonoids in the tomato fruits. Compared with the control, supplementing light for 3 h in the morning significantly increased the contents of six phenolic acids and one flavonoid in the tomato fruit, including *p*-hydroxybenzoic acid, caffeic acid, cynarin, cinnamic acid, benzoic acid, ferulic acid, and quercetin, and the contents of three phenolic acids and one flavonoid: gentisic acid, 4-coumaric acid, gallic acid, and rutin decreased significantly. Supplementing light for 3 h in the morning had no significant effect on the content of three phenolic acids and two flavonoids: protocatechuic acid, chlorogenic acid, sinapic acid, kaempferol, and naringenin. Supplementing light for 3 h at night significantly increased the contents of four phenolic acids and two flavonoids: *p*-hydroxybenzoic acid, sinapic acid, cinnamic acid, ferulic acid, quercetin, and rutin in tomato fruits, while the contents of four phenolic acids and one flavonoid, caffeic acid, gentisic acid, 4-coumaric acid, gallic acid, and naringenin, decreased significantly. Supplementing light for 3 h at night had no significant effect on the contents of four phenolic acids and one flavonoid: protocatechuic acid, chlorogenic acid, cynarin, benzoic acid, and kaempferol (Figure 6A, see also Supplementary Table 5). Moreover, compared with the control plants, supplementary lighting for 3 h in the morning was found to promote significant accumulations of total phenolic acids in tomato fruits, whereas we detected significant reductions in total flavonoid contents. Contrastingly, there were significant increases in the contents of total flavonoids in tomato fruits in response to 3 h of light supplementation in the evening, although this treatment had no significant effects on the accumulation of total phenolic acids (Figures 6B,C).

### Mineral Contents

Table 1 shows the contents of minerals in tomato fruits under different light supplementation periods. Compared with the control, supplementing light for 3 h in the morning significantly increased the accumulation of Ca in tomato fruit, but significantly decreased the contents of P, Cu, Fe and Zn in tomato fruit, and had no significant effect on the contents of K, Mg, Na and Mn. Supplementing light for 3 h in the evening significantly increased the accumulation of Ca in tomato fruit but decreased the content of P and Zn, and there was no significant difference in the content of K, Mg, Na, Cu, Fe, and Mn.

**Table 1:** Mineral contents in tomato fruits under different LED supplementary light periods (mg/g).

**Treatments**	**P**	**K**	**Ca**	**Mg**	**Na**	**Cu**	**Fe**	**Mn**	**Zn**
CK	8.24 ± 0.30^a^	55.13 ± 5.45^a^	1.31 ± 0.04^b^	1.47 ± 0.09^a^	0.089 ± 0.013^a^	0.334 ± 0.076^a^	0.159 ± 0.022^a^	0.0246 ± 0.003^a^	0.0856 ± 0.009^a^
T1	7.52 ± 0.33^b^	50.25 ± 2.88^a^	1.53 ± 0.03^a^	1.60 ± 0.04^a^	0.067 ± 0.002^a^	0.199 ± 0.014^b^	0.121 ± 0.005^b^	0.0252 ± 0.004^a^	0.0433 ± 0.008^b^
T2	7.31 ± 0.17^b^	51.05 ± 3.62^a^	1.60 ± 0.05^a^	1.49 ± 0.08^a^	0.072 ± 0.006^a^	0.236 ± 0.076^ab^	0.148 ± 0.016^ab^	0.0256 ± 0.004^a^	0.0567 ± 0.009^b^

*The data are expressed as average values (n = 3). Different lowercase letters indicate significant differences between treatments (P < 0.05) according to Duncan's multiple range test. CK, no light supplementation control; T1, light supplementation for 3 h in the morning; T2, light supplementation for 3 h in the evening*.

### Differences in the Aroma Characteristics of Tomato Fruits

**Figure 7:**
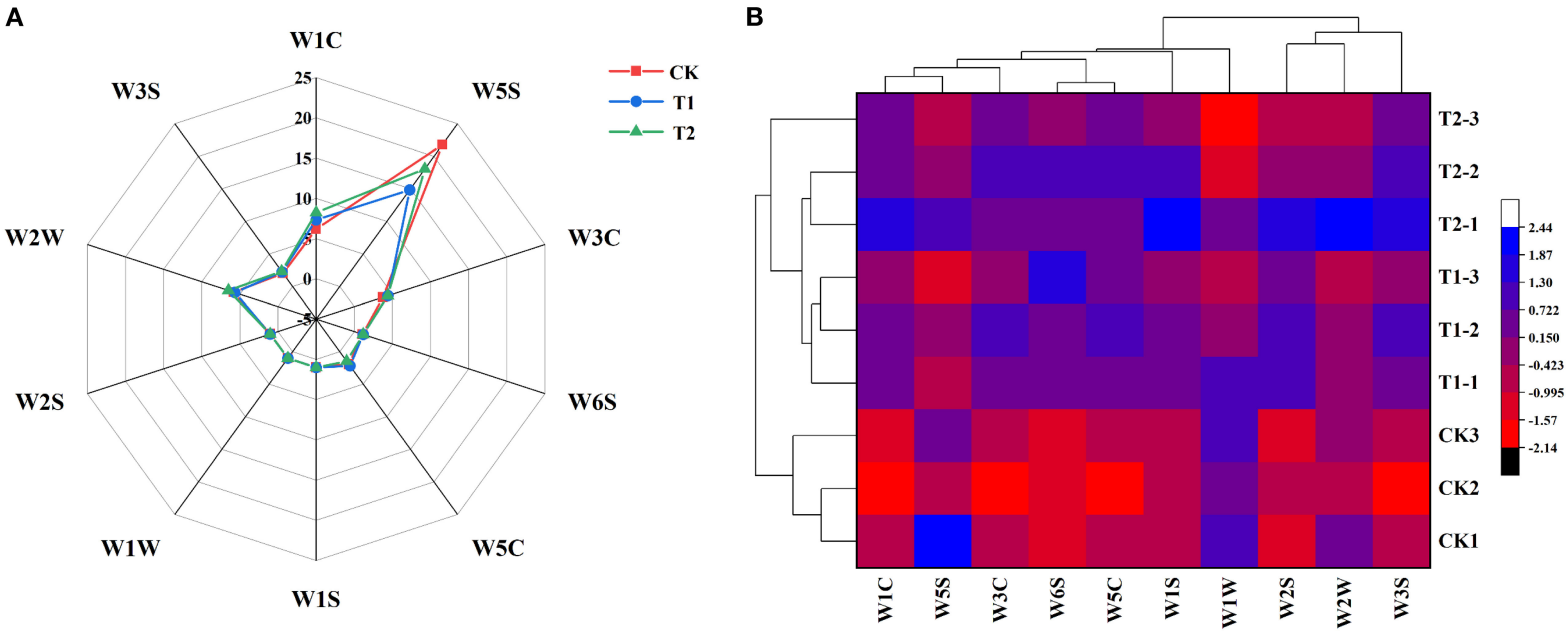
Aroma characteristic response values **(A)** and HCA **(B)** of tomato fruits under different LED supplementary light periods. The data are expressed as average values (*n* = 3). CK, no light supplementation control; T1, light supplementation for 3 h in the morning; T2, light supplementation for 3 h in the evening.

In order to study the effect of supplementary light period on the aroma characteristics of tomato, we used electronic nose (E-nose) to analyze the volatile compounds of tomato fruit. The radar map presented in Figure 7A shows the difference in the response values of the 10 sensors of the E-nose to volatile compounds in tomato fruits exposed to the different treatments. We found that the W5S, W1C, W2W, W3C, and W3S sensors, which are specifically responsive to nitrogen oxides, aromatic benzene compounds, aromatic organic sulfide substances, aromatic ammonia compounds, and long-chain alkanes, respectively, were more sensitive to the volatile compounds in tomato fruits. We also carried out hierarchical cluster analysis (Figure 7B) on the aroma characteristics of tomato fruits exposed to different supplementary light treatments. We accordingly found that the three treatments clustered into two categories, with the control treatment without supplementary lighting comprising one cluster, and the 3 h morning and evening supplementary light treatments comprising the other. This result further indicated that the supplementary light treatment changed the aroma components of tomato fruits. Supplementary light for 3 h in the morning increased the contents of ammonia aromatics and long chain alkanes in the tomato fruits, while light supplementation for 3 h in the evening increased the contents of ammonia aromatics, long chain alkanes, and benzene aromatics in the tomato fruits (Supplementary Table 6).

## Discussion

With the rapid development of LED light supplement technology, its use to improve the quality of vegetables and fruits has attracted research attention (40–42). We used red and blue (7R2B) LEDs to supplement the light of the flowering tomatoes in the morning and evening and analyzed the differences in the quality of tomatoes with different treatments using metabolomics-related technology. Sugars and organic acids in tomato fruit are the key components that affect tomato quality and people's favorite degree of flavor, so appropriately increasing the contents of sugar and organic acid in tomato fruit can improve the quality of tomatoes (43). Our study found that light supplementation for 3 h in the evening promoted the accumulation of fructose, glucose, sucrose, and total sugar in tomato fruit, while light supplementation for 3 h in the morning had no significant effect on the contents of fructose, glucose, and total sugar. However, light supplementation for 3 h in the morning significantly reduced the content of sucrose in the fruit, because there was no significant change in the content of total sugar in the fruit (Figure 2). This may be because the supplementary light in the morning promoted the decomposition of sucrose in tomato fruit to some extent and accelerated the conversion of sucrose to fructose and glucose (44). The study of organic acid in tomato fruit showed that supplementing light for 3 h in the morning increased the contents of tartaric acid, ascorbic acid, oxalic acid, and total organic acid in tomato fruit, but had no significant effect on the accumulation of malic acid and citric acid. Supplementing light for 3 h at night increased the content of tartaric acid and decreased the content of citric acid in tomato fruit but had no significant effect on the contents of malic acid, ascorbic acid, oxalic acid, and total organic acid (Figure 3). The sour taste of tomato is mainly attributed to citric acid and malic acid, and citric acid accounts for the highest proportion of the organic acids in tomato fruit (45). Thus, these results showed that supplementing light for 3 h in the evening significantly reduced the sour taste of tomato fruit, while supplementing light for 3 h in the morning increased the sour taste of tomato fruit. To sum up, supplementing light for 3 h in the evening can increase the content of soluble sugar in tomato fruit and reduce the content of organic acid, improve the flavor quality of tomato fruit, and may enhance the favorite degree of consumers.

Amino acids are the basic substances that make up proteins, which can maintain the normal metabolism of life and provide material basis for life activities. Amino acids have rich tastes such as sweetness, sourness, bitterness and saltiness, and are important taste substances in tomato fruits (46). Our study found that supplementing light for 3 h in the morning could significantly increase the contents of most amino acids and total amino acids in tomato fruits, while supplementing light for 3 h in the evening could also increase the contents of some amino acids and total amino acids in tomato fruits. However, the type and degree of improvements were significantly weaker than those following light supplement for 3 h in the morning (Figure 4). These results showed that supplementing light for 3 h in the morning and 3 h in the evening promoted the accumulation of amino acids in tomato fruits, but the accumulation effect of 3 h in the morning was better, which was similar to the effect of blue light LED irradiation on the content of amino acids in tomato fruits (32).

Carotenoid is the main pigment of tomato fruit and the precursor of odor and flavor synthesis (47). Carotenoid is also a substance that promotes human health and has a good effect on reducing coronary heart disease and lung cancer (48). Our study found that light supplementation for 3 h in the morning promoted the accumulation of beta carotene, lycopene, and total carotenoids in tomato fruits. Light supplementation for 3 h in the evening promoted the accumulation of octahydrolycopene, beta carotene, lycopene, and total carotenoids in tomato fruits, but the increase in the contents of beta carotene and total carotenoids in tomato fruits after light supplementation for 3 h in the evening was weaker than that in the morning. However, the two supplementary light treatments had no significant effect on the accumulation of lutein in fruit (Figure 5). This is similar to the results of Dannehl et al. (26) and Panjai et al. (49); they treated tomatoes with full spectrum LED and red-light LED, respectively, and found that lycopene and beta carotene in tomato fruit increased significantly after the treatments with LEDs.

Phenolic acids and flavonoids are important substances that regulate the sensory quality of vegetables and fruits (50). They have attracted wide attention because of their physiological effects such as antioxidation, anti-inflammation, and anti-allergy; these are important substances that can promote human health (51, 52). Among the 12 phenolic acids and 4 flavonoids we determined, supplementing light for 3 h in the morning increased the accumulation of 6 phenolic acids and total phenolic acids in the tomato fruits but decreased the accumulation of total flavonoids. Supplementing light for 3 h at night increased the accumulation of total flavonoids in tomato fruit but had no significant effect on the accumulation of total phenolic acid (Figure 6). These results showed that supplementing light for 3 h in the morning and 3 h at night had different effects on the accumulation of phenolic acids and flavonoids in tomato fruits, which was similar to results from previous studies. Previous studies found that supplementing light with white light and blue light LED could significantly increased the accumulation of phenols in okra (53), and red-blue light and red-blue-green light LED treatment significantly increased the accumulation of total phenols and flavonoids in tea callus (54).

We also studied the mineral content in tomato fruit. Minerals not only regulate the growth and development of plants, but also play an important role in human health and growth (55, 56). Our study showed that light supplementation for 3 h in the morning and 3 h in the evening promoted the accumulation of Ca in tomato fruits, but light supplementation for 3 h in the morning decreased the contents of P, Cu, Fe, and Zn in tomato fruits, and light supplementation for 3 h in the evening decreased the contents of K, Mg, Na, Cu, Fe, and Mn (Table 1). Some studies have found that red-blue light LED can promote the ripening of tomato fruit by increasing the content of melatonin in tomato fruit (27). In the process of tomato fruit ripening, some mineral content in tomato fruit will decrease to a certain extent (57). In this study, the change of mineral content in tomato fruit may be due to the fact that LED light supplementation promoted the ripening of tomato fruit, which led to the decrease of mineral content in advance of that in fruit without light supplement. Therefore, the mineral content in tomato fruit decreased in different degrees under different light supplement treatments.

Volatile aroma, along with sugars and organic acids, is an important factor affecting tomato fruit flavor, and aroma characteristics are important indicators to distinguish different vegetables and fruits (58). Our study showed that light supplementation for 3 h in the morning and evening significantly increased the contents of ammonia aromatics and long-chain alkanes in tomato fruits, and light supplementation for 3 h in the evening also increased the content of benzene aromatics in tomato fruits. The aroma characteristics of tomato fruits of 3 h in the morning and 3 h in the evening were similar and were clustered into the same category, while the aroma characteristics of tomato fruits without supplemental light treatment were clustered into a separate category (Figure 7). This indicated that the aroma characteristics of tomato fruits were changed by supplementing light for 3 h in the morning and evening, and the benzene aromatic substances in tomato fruits were increased by supplementing light for 3 h in the evening. The latter makes the aroma of tomato fruit more rich (59), which may be more treasured by consumers.

## Conclusion

**Figure 8:**
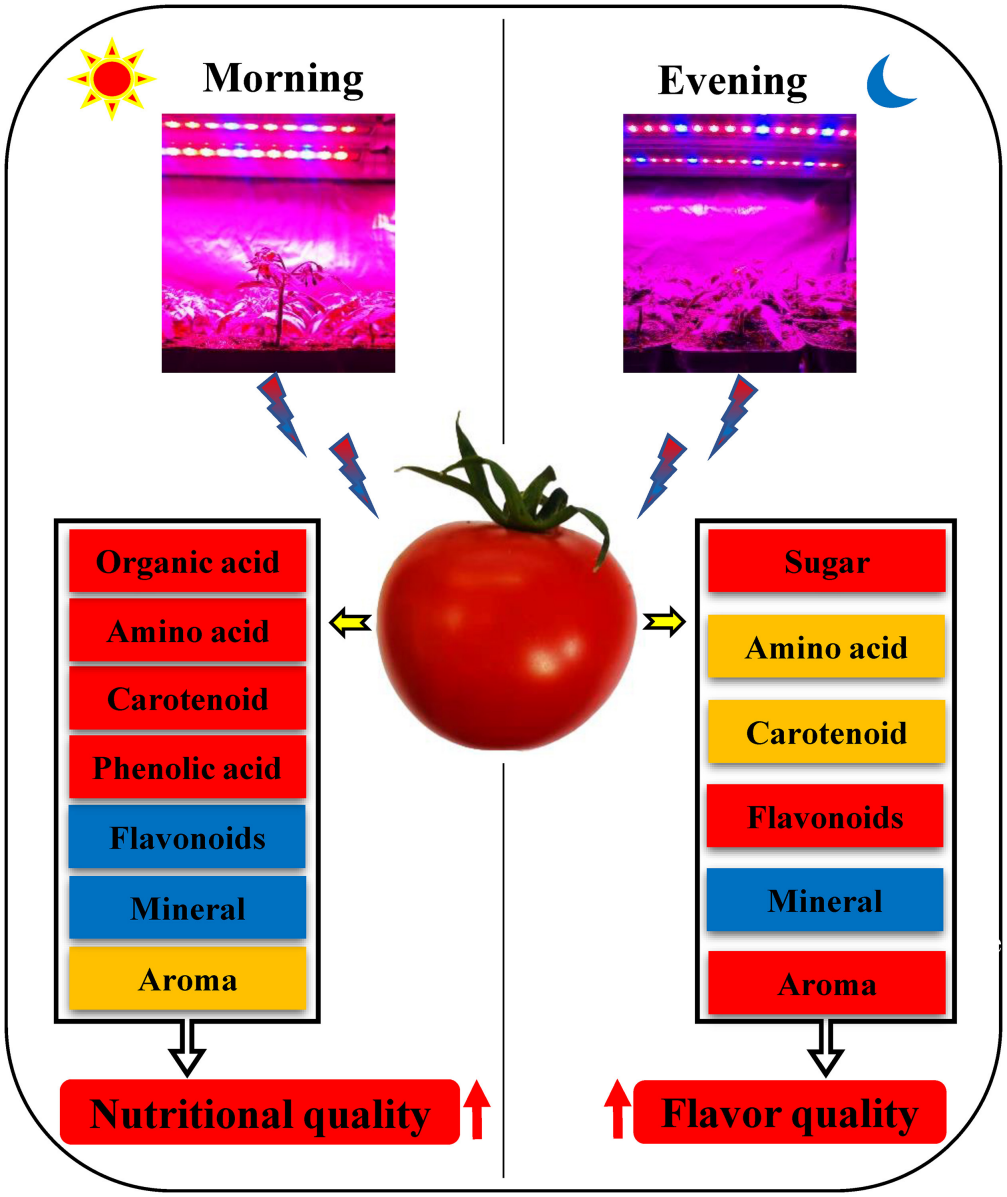
Effects of LED supplementation on fruit quality of tomato in the morning and evening. Red represents an increase in the substance content, orange also represents an increase in the substance content, but the increase is weaker than the red box, and blue represents a decrease in the substance content.

The use of red and blue (7R2B) LEDs to supplement the light of the flowering tomato plants in the morning and evening had different effects on the flavor and nutritional quality of the tomato fruit. Supplementing light for 3 h in the morning significantly increased VC, total organic acids, amino acids, carotenoids, phenolic acids, ammonia aromatics, and long-chain alkanes in the fruit aroma of tomato fruit, and reduced the contents of flavonoids and some minerals, without significant effects on the sugar content of the fruit. Supplementing light for 3 h in the evening significantly increased the contents of sugars, amino acids, carotenoids, flavonoids in tomato fruit, and ammonia aromatic substances, long-chain alkanes, and benzene aromatic substances in the fruit aroma, and decreased the content of minerals, but had no significant effect on the contents of total organic acids and phenolic acids in tomato fruits. However, compared with 3 h of light supplementation in the evening, 3 h of light supplementation in the morning promoted the accumulation of health-promoting substances such as VC, amino acids and carotenoids in tomato fruits. In conclusion, supplementing light for 3 h in the morning improved the nutritional quality of tomato fruit, while supplementing light for 3 h in the evening improved the flavor quality (Figure 8).

## Data Availability Statement

The original contributions presented in the study are included in the article/Supplementary Material, further inquiries can be directed to the corresponding author/s.

## Author Contributions

JL, JY, and SW contributed to conception and design of the study. SW, YX, and WZ participated in the experiment. NJ, LJ, and LH organized the database. SW, NJ, XX, and ZL performed the statistical analysis. SW wrote the first draft of the manuscript. JL, JY, and YW reviewed and revised the manuscript. All authors contributed to the article and approved the submitted version.

## Funding

This research was funded by the Education science and technology innovation project of Gansu Province (GSSYLXM-02), the Special project of central government guiding local science and technology development (ZCYD-2021-07), Gansu people's livelihood science and technology project (20CX9NA099), the Fuxi Young Talents Fund of Gansu Agricultural University (GAUfx-04Y03), the Special project of national modern agricultural industrial system (CARS-23-C-07), and the Gansu excellent postgraduates Innovation Star project (2021CXZX-374).

## Conflict of Interest

The authors declare that the research was conducted in the absence of any commercial or financial relationships that could be construed as a potential conflict of interest.

## Publisher's Note

All claims expressed in this article are solely those of the authors and do not necessarily represent those of their affiliated organizations, or those of the publisher, the editors and the reviewers. Any product that may be evaluated in this article, or claim that may be made by its manufacturer, is not guaranteed or endorsed by the publisher.
