# Supplementary material for: Response of Tomato Fruit Quality Depends on Period of LED Supplementary Light
Source: Front Nutr. 2022 Jan 31;9:833723. doi: 10.3389/fnut.2022.833723 (PMC8841748; doi:10.3389/fnut.2022.833723)
Supplement: Supplementary file 1 [file Presentation_1.pdf]

Supplementary Material

Supplementary Material

Supplementary Table 1. Mass spectrometry parameters for each amino acid

Amino acids	Retention time (min)	Parent ion (m/z)	Daughter ion				
			Quantitative ion (m/z)	Collision energy (V)	Qualitative ion (m/z)	Collision energy (V)	Fragmentor (V)
Phenylalanine	2.95	166.1	120.1	13	103	29	80
Leucine	3.38	132.1	86.1	9	30.2	17	75
Tryptophan	3.41	205.1	188.0	8	146	20	80
Isoleucine	3.75	132.1	86.1	9	44.2	25	75
Methionine	4.22	150.1	104.0	9	56.1	17	75
Valine	4.95	118.1	72.1	9	55.1	25	70
Proline	4.96	116.1	70.1	17	43.2	37	75
Tyrosine	5.01	182.1	136.1	13	91.1	33	85
Cysteine	5.63	122.0	59.1	29	76	13	65
Alanine	6.61	90.1	44.2	9	45.3	40	40
Threonine	6.72	120.1	74.1	9	56.1	17	75
Homoserine	6.91	120.1	74.1	9	56.1	21	70
Glycine	7.00	76.0	30.3	12	NA	NA	35
Glutamine	7.23	147.1	84.1	17	130.1	9	80
Serine	7.26	106.1	88.1	8	42.2	24	67
Asparagine	7.31	133.1	87.1	5	74	17	75
Glutamate	7.68	148.1	84.1	17	130	5	75
Citrulline	7.89	176.1	159.1	9	70.1	25	80
Aspartic acid	8.38	134.0	88.1	9	74	13	70
Histidine	9.06	156.1	110.1	13	83.1	29	90
Arginine	9.54	175.1	70.1	24	60.1	12	100
Lysine	10.16	147.1	84.1	17	130.1	9	75

Supplementary Table 2. Mobile phase elution gradient in the detection of phenolic acids and flavonoids

Time (min)	Flow velocity (mL·min^{-1})	Mobile phase A (Methanol)	Mobile phase B (1% Ethanoic acid)
-	1.1	5%	95%
30	1.1	30%	70%
40	1.1	40%	60%
45	1.1	50%	50%
50	1.1	70%	30%
55	1.1	40%	60%
57	1.1	5%	95%
60	1.1	5%	95%

Supplementary Table 3. Description of substance types and performance of electronic nose sensors.

Sensors	Substance types	Performance
W1C	aromatic	Aromatic components, benzenes
W5S	broadrange	High sensitivity, sensitive to nitrogen oxides
W3C	aromatic	Sensitive aromatic components, ammonia
W6S	hydrogen	Mainly selective to hydride
W5C	arom-aliph	Aromatic components of short-chain alkanes
W1S	broad-methane	Sensitive to methyl groups
W1W	sulphur-organic	Sensitive to sulfides
W2S	broad-alcohol	Sensitive to alcohols, aldehydes and ketones
W2W	sulph-chlor	Aromatic components, sensitive to organic sulfides
W3S	methane-aliph	Sensitive to long-chain alkanes

Supplementary Table 4. Amino Acid contents in tomato fruits under different LED supplementary light periods (ug/g).

Amino acids	CK	T1	T2
Threonine	11.29 ± 0.88[b]	15.71 ± 0.98[a]	12.87 ± 0.86[b]
Phenylalanine	10.40 ± 0.21[c]	15.80 ± 0.18[a]	12.45 ± 0.21[b]
Leucine	19.72 ± 0.62[c]	51.45 ± 1.00[a]	36.02 ± 1.96[b]
Isoleucine	9.46 ± 0.43[c]	25.75 ± 1.35[a]	20.04 ± 0.51[b]
Asparagine	11.47 ± 3.93[b]	28.73 ± 3.82[a]	16.28 ± 0.88[b]
Tryptophan	6.09 ± 0.20[b]	7.61 ± 0.39[a]	7.30 ± 0.66[a]
Methionine	2.41 ± 0.20[c]	7.26 ± 0.87[a]	5.76 ± 0.75[b]
Valine	12.40 ± 0.89[c]	31.42 ± 0.93[a]	25.47 ± 0.89[b]
Proline	10.29 ± 1.01[c]	44.57 ± 1.52[a]	26.81 ± 1.58[b]
Tyrosine	16.45 ± 2.16[c]	27.92 ± 1.27[a]	22.97 ± 1.55[b]
Cysteine	1.96 ± 0.33[b]	6.32 ± 0.95[a]	1.97 ± 0.50[b]
Alanine	42.52 ± 1.36[c]	102.68 ± 1.14[a]	71.98 ± 3.19[b]
Glycine	5.64 ± 1.34[b]	14.32 ± 2.66[a]	13.65 ± 3.65[a]
Serine	22.03 ± 1.75[a]	28.41 ± 5.63[a]	29.36 ± 3.94[a]
Glutamate	76.54 ± 6.94[b]	98.81 ± 1.87[a]	94.74 ± 3.91[a]
Histidine	109.27 ± 21.05[b]	584.44 ± 96.30[a]	307.74 ± 75.45[b]
Aspartic acid	2435.40 ± 63.63[b]	3246.08 ± 90.97[a]	1515.60 ± 35.44[c]
Arginine	619.04 ± 31.23[c]	1715.45 ± 45.49[a]	1301.80 ± 25.06[b]
Cystine	16.59 ± 0.37[b]	55.06 ± 2.11[a]	13.97 ± 3.27[b]
Glutamine	92.97 ± 6.53[b]	249.99 ± 16.19[a]	237.06 ± 9.84[a]
Lysine	106.35 ± 3.50[b]	270.55 ± 14.60[a]	267.90 ± 9.57[a]
Total amino acids	3638.28 ± 95.77[c]	6628.32 ± 203.63[a]	4047.72 ± 134.16[b]

The data are expressed as average values ± SE (n = 3). [a-c]Indicate significant differences between treatments ($P < 0.05$, Duncan's multiple range test). CK: no light supplementation control, T1: light supplementation for 3 h in the morning, T2: light supplementation for 3 h in the evening.

Supplementary Table 5. Phenolic acids and flavonoids contents in tomato fruits under different LED supplementary light periods (ug/g).

Phenolic acids and flavonoids		CK	T1	T2
Phenolic acids	P-hydroxybenzoic acid	74.188 ± 2.39^{b}	105.04 ± 6.30^{a}	109.29 ± 9.92^{a}
	Protocatechuic acid	49.16 ± 5.12^{a}	62.30 ± 11.78^{a}	53.69 ± 3.62^{a}
	Chlorogenic acid	450.63 ± 45.70^{a}	494.04 ± 36.71^{a}	461.56 ± 6.51^{a}
	Sinapic acid	8.68 ± 1.54^{b}	11.23 ± 0.66^{ab}	13.304 ± 2.31^{a}
	Caffeic acid	130.96 ± 3.72^{b}	173.10 ± 3.63^{a}	121.14 ± 3.14^{c}
	Cynarin	14.07 ± 1.23^{b}	18.37 ± 2.67^{a}	15.98 ± 0.26^{ab}
	Gentisic acid	27.59 ± 1.90^{a}	9.91 ± 1.35^{b}	9.51 ± 1.05^{b}
	Cinnamic acid	6.45 ± 1.64^{c}	16.96 ± 2.91^{a}	12.65 ± 0.97^{b}
	4 - coumaric acid	23.38 ± 1.96^{a}	19.17 ± 2.25^{b}	10.22 ± 1.79^{c}
	Gallic acid	262.83 ± 1.29^{a}	183.13 ± 5.24^{c}	227.55 ± 8.71^{b}
	Benzoic acid	493.72 ± 1.43^{b}	576.60 ± 15.82^{a}	511.80 ± 59.49^{ab}
	Ferulic acid	5.89 ± 0.43^{c}	9.37 ± 0.38^{b}	17.73 ± 1.35^{a}
Flavonoids	Quercetin	85.28 ± 4.79^{c}	178.85 ± 10.67^{a}	133.23 ± 13.19^{b}
	Rutin	1400.82 ± 88.47^{b}	924.19 ± 72.08^{c}	2055.81 ± 39.47^{a}
	Kaempferol	6.95 ± 0.54^{a}	7.09 ± 0.97^{a}	7.09 ± 1.02^{a}
	Naringenin	37.41 ± 1.03^{a}	38.14 ± 1.30^{a}	31.82 ± 2.80^{b}

The data are expressed as average values ± SE (n = 3). $^{a\text{-}c}$Indicate significant differences between treatments ($P < 0.05$, Duncan's multiple range test). CK: no light supplementation control, T1: light supplementation for 3 h in the morning, T2: light supplementation for 3 h in the evening.

Supplementary Table 6. Aroma characteristic response values of tomato fruits under different LED supplementary light periods

Sensors	**CK**	**T1**	**T2**
W1C	6.187 ± 0.584^{b}	7.320 ± 0.429^{ab}	8.225 ± 0.791^{a}
W5S	21.764 ± 3.766^{a}	14.852 ± 2.502^{a}	18.064 ± 3.746^{a}
W3C	3.758 ± 0.305^{b}	4.416 ± 0.230^{a}	4.405 ± 0.125^{a}
W6S	1.107 ± 0.004^{b}	1.171 ± 0.018^{a}	1.163 ± 0.018^{a}
W5C	1.929 ± 0.134^{a}	2.181 ± 0..079^{a}	1.462 ± 1.267^{a}
W1S	1.014 ± 0.001^{b}	1.020 ± 0.003^{ab}	1.024 ± 0.007^{a}
W1W	1.013 ± 0.001^{a}	1.008 ± 0.006^{a}	1.003 ± 0.006^{a}
W2S	1.018 ± 0.002^{b}	1.027 ± 0.002^{a}	1.024 ± 0.006^{ab}
W2W	5.794 ± 0.576^{a}	5.662 ± 0.384^{a}	6.495 ± 1.348^{a}
W3S	2.034 ± 0.069^{b}	2.241 ± 0.082^{a}	2.319 ± 0.063^{a}

The data are expressed as average values ± SE (n = 3). $^{a\text{-}b}$Indicate significant differences between treatments ($P < 0.05$, Duncan's multiple range test). CK: no light supplementation control, T1: light supplementation for 3 h in the morning, T2: light supplementation for 3 h in the evening.
